# Aortic flow is associated with aging and exercise capacity

**DOI:** 10.1093/ehjopen/oead079

**Published:** 2023-08-26

**Authors:** Xiaodan Zhao, Pankaj Garg, Hosamadin Assadi, Ru-San Tan, Ping Chai, Tee Joo Yeo, Gareth Matthews, Zia Mehmood, Shuang Leng, Jennifer Ann Bryant, Lynette L S Teo, Ching Ching Ong, James W Yip, Ju Le Tan, Rob J van der Geest, Liang Zhong

**Affiliations:** National Heart Research Institute Singapore, National Heart Centre Singapore, 5 Hospital Drive, 169609 Singapore, Singapore; Cardiology Department, Norfolk and Norwich University Hospitals NHS Foundation Trust, Colney Ln, Norwich, NR4 7UY Norfolk, UK; Department of Cardiovascular and Metabolic Health, Norwich Medical School, University of East Anglia, Rosalind Franklin Rd, Norwich, NR4 7UQ Norfolk, UK; Cardiology Department, Norfolk and Norwich University Hospitals NHS Foundation Trust, Colney Ln, Norwich, NR4 7UY Norfolk, UK; Department of Cardiovascular and Metabolic Health, Norwich Medical School, University of East Anglia, Rosalind Franklin Rd, Norwich, NR4 7UQ Norfolk, UK; National Heart Research Institute Singapore, National Heart Centre Singapore, 5 Hospital Drive, 169609 Singapore, Singapore; Duke-NUS Medical School, National University of Singapore, 8 College Road, 169857 Singapore, Singapore; Department of Diagnostic Imaging, National University Hospital Singapore, 5 Lower Kent Ridge Road, 119074 Singapore, Singapore; Yong Loo Lin School of Medicine, National University of Singapore, 10 Medical Drive, 117597 Singapore, Singapore; Department of Diagnostic Imaging, National University Hospital Singapore, 5 Lower Kent Ridge Road, 119074 Singapore, Singapore; Yong Loo Lin School of Medicine, National University of Singapore, 10 Medical Drive, 117597 Singapore, Singapore; Cardiology Department, Norfolk and Norwich University Hospitals NHS Foundation Trust, Colney Ln, Norwich, NR4 7UY Norfolk, UK; Department of Cardiovascular and Metabolic Health, Norwich Medical School, University of East Anglia, Rosalind Franklin Rd, Norwich, NR4 7UQ Norfolk, UK; Cardiology Department, Norfolk and Norwich University Hospitals NHS Foundation Trust, Colney Ln, Norwich, NR4 7UY Norfolk, UK; Department of Cardiovascular and Metabolic Health, Norwich Medical School, University of East Anglia, Rosalind Franklin Rd, Norwich, NR4 7UQ Norfolk, UK; National Heart Research Institute Singapore, National Heart Centre Singapore, 5 Hospital Drive, 169609 Singapore, Singapore; Duke-NUS Medical School, National University of Singapore, 8 College Road, 169857 Singapore, Singapore; National Heart Research Institute Singapore, National Heart Centre Singapore, 5 Hospital Drive, 169609 Singapore, Singapore; Duke-NUS Medical School, National University of Singapore, 8 College Road, 169857 Singapore, Singapore; Department of Diagnostic Imaging, National University Hospital Singapore, 5 Lower Kent Ridge Road, 119074 Singapore, Singapore; Yong Loo Lin School of Medicine, National University of Singapore, 10 Medical Drive, 117597 Singapore, Singapore; Department of Diagnostic Imaging, National University Hospital Singapore, 5 Lower Kent Ridge Road, 119074 Singapore, Singapore; Yong Loo Lin School of Medicine, National University of Singapore, 10 Medical Drive, 117597 Singapore, Singapore; Department of Diagnostic Imaging, National University Hospital Singapore, 5 Lower Kent Ridge Road, 119074 Singapore, Singapore; Yong Loo Lin School of Medicine, National University of Singapore, 10 Medical Drive, 117597 Singapore, Singapore; National Heart Research Institute Singapore, National Heart Centre Singapore, 5 Hospital Drive, 169609 Singapore, Singapore; Duke-NUS Medical School, National University of Singapore, 8 College Road, 169857 Singapore, Singapore; Department of Radiology, Leiden University Medical Center, Albinusdreef 2, 2333 ZA Leiden, TheNetherlands; National Heart Research Institute Singapore, National Heart Centre Singapore, 5 Hospital Drive, 169609 Singapore, Singapore; Duke-NUS Medical School, National University of Singapore, 8 College Road, 169857 Singapore, Singapore

**Keywords:** Flow displacement, Aortic flow, 2D phase-contrast, Cardiopulmonary exercise testing, Haemodynamics

## Abstract

**Aims:**

Increased blood flow eccentricity in the aorta has been associated with aortic (AO) pathology, however, its association with exercise capacity has not been investigated. This study aimed to assess the relationships between flow eccentricity parameters derived from 2-dimensional (2D) phase-contrast (PC) cardiovascular magnetic resonance (CMR) imaging and aging and cardiopulmonary exercise test (CPET) in a cohort of healthy subjects.

**Methods and Results:**

One hundred and sixty-nine healthy subjects (age 44 ± 13 years, M/F: 96/73) free of cardiovascular disease were recruited in a prospective study (NCT03217240) and underwent CMR, including 2D PC at an orthogonal plane just above the sinotubular junction, and CPET (cycle ergometer) within one week. The following AO flow parameters were derived: AO forward and backward flow indexed to body surface area (FFi, BFi), average flow displacement during systole (FDs_avg_), late systole (FDls_avg_), diastole (FDd_avg_), systolic retrograde flow (SRF), systolic flow reversal ratio (sFRR), and pulse wave velocity (PWV). Exercise capacity was assessed by peak oxygen uptake (PVO_2_) from CPET. The mean values of FDs_avg_, FDls_avg_, FDd_avg_, SRF, sFRR, and PWV were 17 ± 6%, 19 ± 8%, 29 ± 7%, 4.4 ± 4.2 mL, 5.9 ± 5.1%, and 4.3 ± 1.6 m/s, respectively. They all increased with age (*r* = 0.623, 0.628, 0.353, 0.590, 0.649, 0.598, all *P* < 0.0001), and decreased with PVO_2_ (r = −0.302, −0.270, −0.253, −0.149, −0.219, −0.161, all *P* < 0.05). A stepwise multivariable linear regression analysis using left ventricular ejection fraction (LVEF), FFi, and FDs_avg_ showed an area under the curve of 0.769 in differentiating healthy subjects with high-risk exercise capacity (PVO_2_ ≤ 14 mL/kg/min).

**Conclusion:**

AO flow haemodynamics change with aging and predict exercise capacity.

**Registration:**

NCT03217240

## Graphical Abstract

**Graphical abstract:**
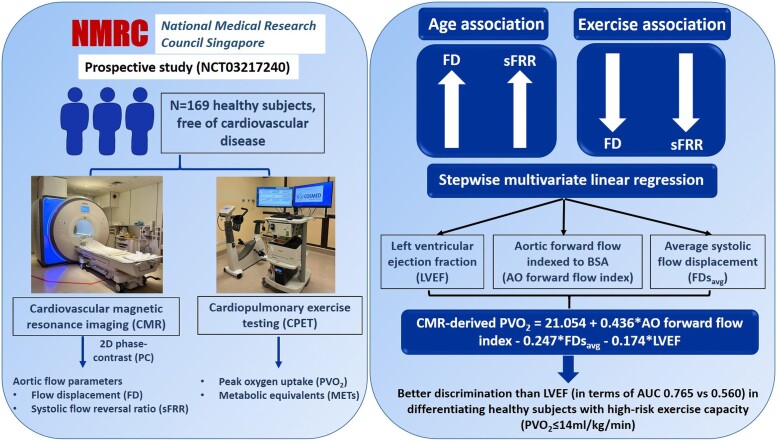

## Introduction

Aortic (AO) flow is essential for delivering oxygenated blood to all organs and tissues of the body, making it crucial for maintaining overall health and functioning. AO flow patterns have exceptional adaptability to maintain normal blood circulation under a broad range of haemodynamic workloads. In healthy individuals, the ascending AO systolic flow is spiralling forward.^1^ This occurs efficiently with a central laminar profile that minimizes resistance and distributes wall stress circumferentially and homogeneously.^2^ It is well-recognized that the flow in the aorta is not purely pulsatile and axial. A degree of helicity exists in normal ascending AO flow as blood exits the AO valve anteriorly towards the right wall of the aorta and then propagates posterolaterally, creating a dominant, larger right-handed helix and a smaller concurrent left-handed helix, usually with less than 180° rotation.

It is well-established that aging is associated with reduced exercise capacity and breathlessness. Moreover, aging is also associated with significant changes in the AO biomechanics, particularly increasing vascular stiffness.^3^ Due to increased vascular stiffness, blood pressure rises with age, possibly leading to AO root dilatation.^4,5^ AO flow haemodynamics have been shown to be significantly changed with advancing age using four-dimensional (4D) flow cardiovascular magnetic resonance (CMR).^6,7,8,9,10^ 4D flow CMR can provide flow visualization and quantification in three directions. It is not routinely acquired in standard CMR examinations. The association between aging and AO flow haemodynamics as assessed using 4D flow CMR, or indeed two-dimensional (2D) phase-contrast (PC) CMR, is less well studied, and more importantly, the inter-relationship with exercise capacity remains unknown. The present study hypothesizes that increasing age is associated with abnormal metrics of AO flow eccentricity, which, in turn, result in quantitative reductions in exercise capacity as assessed by cardiopulmonary exercise testing (CPET). The main objective of this study was to evaluate ascending AO flow haemodynamics using 2D PC CMR imaging methods and investigate their association with age and peak oxygen uptake (PVO_2_) and metabolic equivalents (METs) on CPET in healthy controls of different ages.

## Methods

### Study population

One hundred eighty-five healthy subjects aged twenty to eighty were identified from a prospective multicentre registry (NCT03217240) that recruited both patients and healthy subjects without known cardiovascular disease or cardiovascular risk factors (hypertension, diabetes, and hyperlipidaemia). Only the latter were analysed in this paper. Of these, 169 healthy subjects who had undergone 2D PC CMR and CPET (within one week of the CMR scan) were included in the final analysis. Subjects were further stratified into three age groups based on the 25th, 50th, and 75th percentiles: Group one (*n* = 58, M/F: 35/23, age ranges: 21–36 years), Group two (*n* = 56, M/F: 30/26, age ranges: 37–50 years), and Group three (*n* = 55, M/F: 31/24, age ranges: 51–76 years). This study had been approved by the Institutional Review Boards, and written informed consent was obtained from each subject.

### Cardiac magnetic resonance protocol

CMR acquisition was performed on 3.0T Ingenia (Philips Healthcare, Best, the Netherlands) and 1.5T Magneton Aera (Siemens Healthineers, Erlangen, Germany) scanners, as previously published.^11,12,13^ Balanced steady-state free precession end-expiratory breath-hold cine images were acquired for the two-, three-, and four-chamber long-axis and a stack of short-axis images covering the entire left ventricle (LV) and right ventricle (RV) and reconstructed with a temporal resolution of 30 frames per heart cycle. Axial scout images with a bright-blood sequence for anatomy were also acquired. 2D PC magnitude and velocity images of the ascending and descending aorta were obtained in a single transverse plane that transected the ascending aorta at the level of the right pulmonary artery. The detailed acquisition parameters for both cine and 2D PC images for two scanners are provided in Supplementary material online, *Table S1*.

### Cardiac magnetic resonance image analysis

All CMR image analyses were performed at a core laboratory using MASS research software (Version 2022-EXP, Leiden University Medical Center, Leiden, The Netherlands).

### Biventricular function measurement and aortic measurements

Artificial intelligence (AI)-based automatic segmentation of LV and RV endocardial and epicardial borders in the short-axis stacks was performed to generate volume curves throughout the cardiac cycle.^14^ In our study cohort, manual adjustments of AI segmented contours, typically in the apical slices, were performed in 32/169 (18.9%) cases after visually reviewing the contours. Papillary and trabecular muscles were included in the volume calculation. End-diastolic volume (EDV) and end-systolic volume (ESV) were defined respectively as maximal and minimal values of the volume curve. LV mass was estimated at end-diastole. LV mass and all volumetric parameters were indexed to body surface area (BSA). In the left ventricular outflow tract (LVOT) view at the end-diastolic phase, the AO dimeters at the levels of AO valve annulus, the widest point of the AO sinuses, and the sinotubular junction were measured.^15^

### Two-dimensional phase-contrast flow analysis

**Figure 1:**
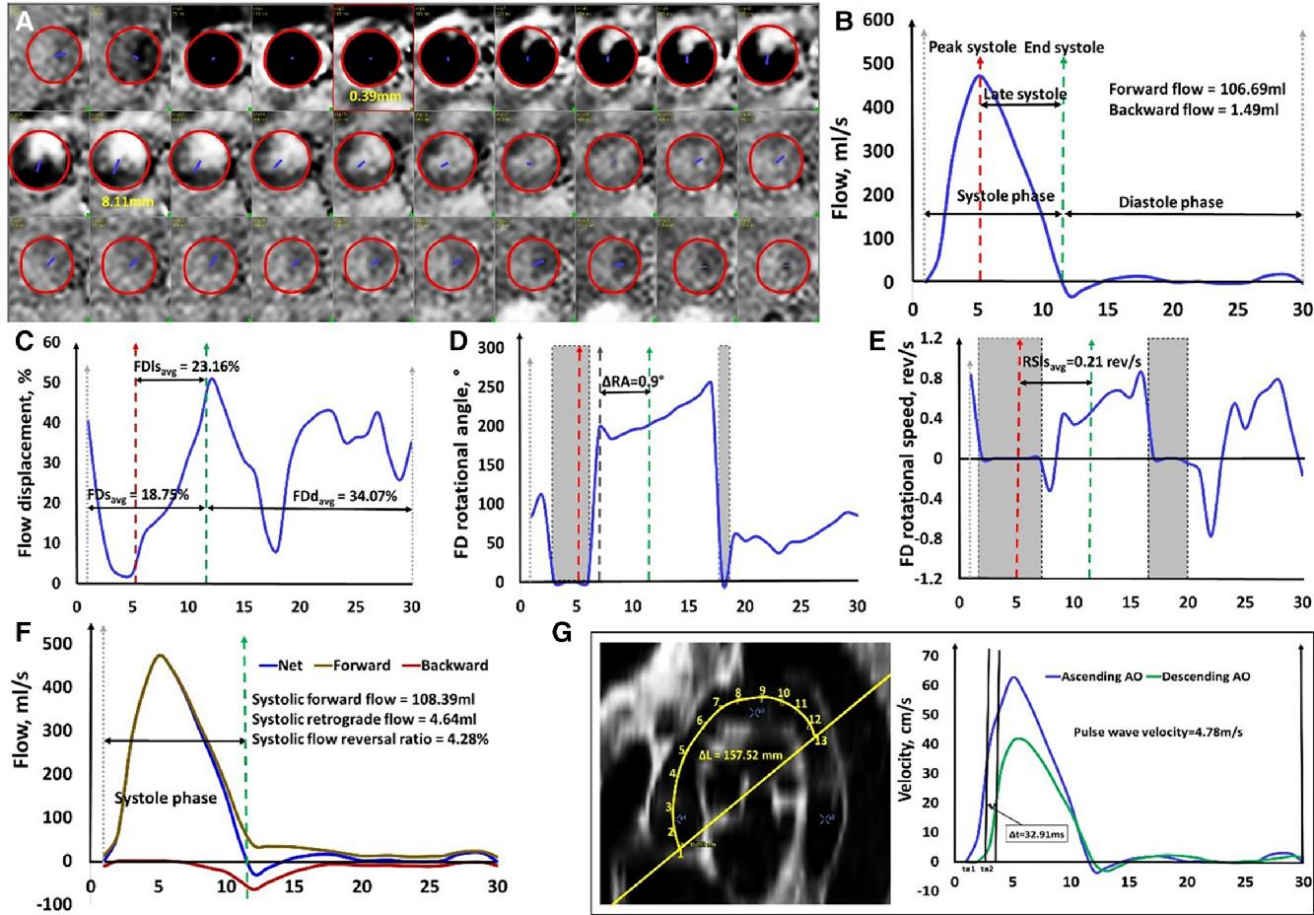
Illustration of two-dimensional aortic flow parameter calculations. (*A*) Segmentation of aorta for whole cardiac cycle in two-dimensional phase-contrast magnetic resonance imaging. The borders were segmented on the reconstructed magnitude images and copied onto the phase images; (*B*) Aortic flow curve with illustrations of peak systole, late systole, systole, and diastole phase; (*C*) Flow displacement curve; (*D*) Flow displacement rotational angle curve; (*E*) Flow displacement rotational speed curve; (*F*) Flow reversal ratio curve; (*G*) Three-dimensional aortic arch length between ascending and descending aorta by reconstructing the aortic arch using bright-blood sequence, and pulse wave velocity by the half-maximum method. The grey areas denoted flow displacement ≤12% and were excluded in the calculations of rotational angle and rotational speed. The unit of the x-axis in each figure is the frame number.

Semi-automatic segmentation of ascending and descending aorta throughout the cardiac cycle was performed using MASS with manual adjustment where necessary (*Figure 1A*). Note that the segmentations were performed on the magnitude data from the PC images and copied to the phase data for further flow calculations. This approach has previously been shown to be very reproducible.^16^ The following parameters were automatically derived based on the AO contours:

- The maximal and minimal area were selected by computing the cross-section area of ascending aorta during the cardiac cycle, and relative area change (RAC) was calculated as (maximal area−minimal area)/minimal area × 100%.
- Forward and backward flows were obtained from the resultant flow curve (*Figure 1B*), and both were indexed to BSA. The peak systole phase was automatically registered as the phase of peak flow rate on the flow curve. The end systole phase was determined where the downward slope of the descending systolic flow curve intersected the x-axis (or, no flow). Late systole (ls) period was defined as the time window from peak systole to end systole.
- Flow displacement (FD) was calculated as the distance between the vessel centre point and the centre-of-velocity of the forward flow and was normalized to the overall vessel size for each cardiac phase.^17,18^ Average FD during systole (FDs_avg_), late systole (FDls_avg_), and diastole (FDd_avg_) were calculated from the time-resolved FD curve (*Figure 1C*). FD at peak systole (FDps) was also recorded. The computations of center point, centre-of-velocity, and vessel size are given in Supplementary material online, *Extended Methods*.
- FD rotational angle change (ΔRA) was determined as rotational angle (RA) at end systole—RA at the point where the flow angle stabilized after peak systole from the RA curve (*Figure 1D*). A figure illustrating the definition of RA at 0 and 180 degrees and calculation of ΔRA is given in Supplementary material online, *Figure S1* with the definition of RA given in the figure legend. In the current study, the RA was set to 0 if FD ≤12% as the AO blood flow is mainly laminar in early systole.^19^ The detailed methods and explanation of choosing the 12% threshold are given in Supplementary material online, *Extended Methods*.
- FD rotational speed (RS) was indicative of a helical flow pattern as the RA varied from phase to phase. A figure illustrating how to calculate RS at a given phase is presented in Supplementary material online, *Figure S1* with the derivation of RS given in the figure legend. RSls_avg_ was determined as the average RS after peak systole till the end of systole (*Figure 1E*). When FD < 12%, the RS was not computed. It was set to be 0 in the graph, but it was not used in further analysis.
- Systolic flow reversal ratio (sFRR). The forward and retrograde components in the AO flow were separated based on the automated selection of the pixels with the same through-plane velocity sign within each AO section from PC velocity images.^20^ The corresponding flow curves through the whole cardiac cycle were generated, and sFRR was calculated as sFRR (%) = systolic retrograde flow (SRF)/systolic forward flow (SFF)×100 (*Figure 1F*).
- Pulse wave velocity (PWV) was calculated as the ratio of distance and transit time between ascending to descending aorta (*Figure 1G*).^21^ The approach to computing the distance and transit time is given in Supplementary material online, *Extended Methods*.

### Cardiopulmonary exercise testing

All subjects underwent CPET at a central laboratory within one week after CMR. The protocol was provided in our previous publication.^12,13^ Minute ventilation (VE), oxygen consumption (VO_2_), and carbon dioxide output (VCO_2_) were acquired breath-by-breath and averaged over 10-s intervals. One MET was defined as the amount of oxygen consumed while sitting at rest (i.e. 3.5 mL of oxygen per kilogram body weight per minute). Peak oxygen uptake (PVO_2_) was the highest 10-s averaged sample obtained during exercise. % predicted PVO_2_ was calculated based on proposed normative values.^22,23^ VE/VCO_2_ slope was calculated via least squares linear regression (y = mx + b, m = slope) using VE and VCO_2_ values acquired from the start of exercise to peak. We further stratified subjects into three groups: normal exercise capacity (PVO_2_ > 20 mL/kg/min, *n* = 120); low- and intermediate-risk exercise capacity (14 < PVO_2_ ≤ 20 mL/kg/min, *n* = 36,) and high-risk exercise capacity (PVO_2_ ≤ 14 mL/kg/min, *n* = 13).^24^

### Statistical analysis

Data were analysed using SPSS (version 25.0, Chicago, IL, USA). Continuous variables were expressed as mean ± standard deviation (SD) for normally distributed data or median (25th percentile, 75th percentile) for non-normally distributed data. Comparison of means for more than two groups was analysed using one-way analysis of variance (ANOVA) for normally distributed data, and Kruskal–Wallis (K–W) non-parametric one-way ANOVA for more than two groups with post-hoc pair-wise comparisons in the event of a significant K–W test for non-normally distributed data. The χ^2^ test or Fisher’s exact test, as appropriate, was used to analyse categorical variables. Associations between continuous variables were investigated using correlation (Pearson). Univariate and multi-variable stepwise regression analyses were performed to investigate the determinants of PVO_2_. Variables that showed significant associations (*P* < 0.05) on univariate analyses were input as independent variables for multi-variable linear regression analyses, and a regression model was constructed. Receiver operator characteristic (ROC) analyses were performed to assess the discriminative capability of these blood flow parameters to high-risk exercise capacity. Youden’s indexes were defined for all points of the ROC curve and the maximum value was used as the criterion for selecting the optimum threshold point.^25^ A nested binary logistic regression analysis was used to investigate the incremental value of blood flow parameters over LV ejection fraction (LVEF) for discriminating subjects with high-risk exercise capacity. Statistical significance was declared at *P* < 0.05.

To evaluate the reproducibility of AO blood flow parameters, intraobserver, and interobserver reproducibility were assessed on a randomly selected subgroup of 20 subjects using paired *t*-tests, intraclass correlation (ICC) for average measures with two-way mixed model and consistence type, coefficient of variation (CV), and Bland–Altman plots. CV was calculated as the ratio of the SD to the mean between the two measurements, wherein $SD=\sqrt{\frac{\sum {\left(measurement\;1\;-\;measurement\;2\right)}^{2}}{2n}}\;$, $mean\;=\frac{\sum (measurement\;1+\;measurement\;2)}{2n}$, *n* being the number of data pair. During repeated analysis, adjustment to the semi-automatically segmented AO contours were applied when needed, while the downstream AO blood flow parameters were automatically calculated based on these AO contours.

## Results

### Demographic characteristics and baseline cardiovascular magnetic resonance data and aortic flow

The mean age of the healthy subjects was 44 ± 13 years, with 73 (43%) being females. The mean ages of the three age groups were 30 ± 4, 43 ± 4, and 60 ± 5 years, respectively. As expected, Group three had shorter height, higher systolic blood pressure, and significantly smaller indexed LV and RV volumes than Group one (*Table 1*). No differences were observed in gender, weight, diastolic blood pressure, BSA, heart rate, LV mass index, LV ejection fraction (LVEF), and RVEDV/LVEDV ratio among the three age groups, where RVEDV (resp. LVEDV) denoted right (left) ventricular end-diastolic volume. There were significant differences in AO diameters at valve annulus, sinuses, and sinotubular junction among three age groups. The mean values of average FD (FDs_avg_, FDls_avg_, FDd_avg_), peak systolic FD, systolic forward flow (SFF), SRF, systolic flow reverse ratio (sFRR), and PWV were 17 ± 6%, 19 ± 8%, 29 ± 7%, 7 ± 5%, 73.0 ± 14.0 mL, 4.42 ± 4.24 mL, 5.9 ± 5.1%, and 4.3 ± 1.6 m/s, respectively. The median values of PVO_2_, METs, % predicted PVO_2_ and VE/VCO_2_ slope were 24 mL/kg/min, 6.8, 90%, and 26. Compared with male subjects, female subjects had significantly smaller AO backward flow index, AO max and min area and SFF, and larger RAC, FDs_avg_, FDls_avg_, and FDps; in addition, PVO_2_ and METs were also decreased in female subjects (see Supplementary material online, *Table S2*).

**Table 1:** Demographics, 2D aortic flow, and cardiopulmonary exercise test parameters for the overall population and three age subgroups by age quantiles (25th, 50th, and 75th)

	All	Group one	Group two	Group three	*P*
	(*n* = 169)	(*n* = 58)	(*n* = 56)	(*n* = 55)	
**Demographics**					
Age, years	44 ± 13	30 ± 4	43 ± 4*	60 ± 5*^#^	**<0**.**001**
Gender, M/F	96/73	35/23	30/26	31/24	0.764
Weight, kg	65 ± 13	66 ± 14	64 ± 11	64 ± 13	0.737
Height, cm	166 ± 9	169 ± 9	165 ± 9	165 ± 8*	**0**.**020**
Systolic blood pressure, mmHg	126 ± 17	121 ± 17	125 ± 14	132 ± 16*^#^	**0**.**001**
Diastolic blood pressure, mmHg	76 ± 13	74 ± 12	77 ± 11	79 ± 12	0.057
Body surface area, m^2^	1.72 ± 0.19	1.75 ± 0.20	1.71 ± 0.19	1.70 ± 0.19	0.283
Heart rate, bpm	73 ± 13	74 ± 13	74 ± 12	70 ± 13	0.166
**LV function**					
LV mass index, g/m^2^	48 ± 11	49 ± 10	49 ± 14	47 ± 9	0.574
LVEDV index, mL/m^2^	73 ± 13	76 ± 14	69 ± 11*	67 ± 10*	**<0**.**001**
LVESV index, mL/m^2^	26 ± 8	29 ± 9	25 ± 8*	25 ± 7*	**0**.**009**
LVSV index, mL/m^2^	45 ± 7	47 ± 7	44 ± 7	42 ± 6*	**0**.**002**
LV ejection fraction, %	63 ± 7	63 ± 7	64 ± 8	63 ± 7	0.473
**RV function**					
RVEDV index, mL/m^2^	77 ± 15	84 ± 15	74 ± 15*	72 ± 12*	**<0**.**001**
RVESV index, mL/m^2^	34 ± 10	39 ± 11	32 ± 10*	31 ± 9*	**<0**.**001**
RVSV index, mL/m^2^	43 ± 7	46 ± 7	42 ± 7*	41 ± 6*	**0**.**001**
RV ejection fraction, %	57 ± 7	55 ± 6	58 ± 7	57 ± 6	**0**.**034**
RVEDV/LVEDV ratio	1.09 ± 0.11	1.11 ± 0.10	1.07 ± 0.10	1.08 ± 0.12	0.085
AO diameter					
Aortic valve annulus, mm	21.7 ± 2.4	22.4 ± 2.5	21.8 ± 2.3	21.0 ± 2.1*	**0**.**005**
Aortic sinuses, mm	28.8 ± 3.5	27.7 ± 3.4	28.4 ± 3.3	30.4 ± 3.3*^#^	**<0**.**001**
Sinotubular junction, mm	23.4 ± 2.9	22.1 ± 2.6	23.6 ± 3.0*	24.7 ± 2.6*	**<0**.**001**
**2D aortic flow parameters**					
AO forward flow index, mL/m^2^	42.7 ± 6.5	44.5 ± 6.3	42.4 ± 6.7	41.0 ± 6.1*	**0**.**014**
AO backward flow index, mL/m^2^	0.55 ± 0.56	0.33 ± 0.34	0.53 ± 0.58	0.81 ± 0.63*^#^	**<0**.**001**
AO maximal area, cm^2^	7.1 ± 1.7	6.1 ± 1.0	7.1 ± 1.5*	8.3 ± 1.7*^#^	**<0**.**001**
AO minimal area, cm^2^	5.6 ± 1.6	4.6 ± 1.0	5.5 ± 1.2*	7.0 ± 1.7*^#^	**<0**.**001**
Relative area change, %	29 ± 16	36 ± 18	30 ± 15	20 ± 11*^#^	**<0**.**001**
FDs_avg_, %	17 ± 6	12 ± 4	17 ± 4*	21 ± 6*^#^	**<0**.**001**
FDls_avg_, %	19 ± 8	13 ± 6	19 ± 6*	26 ± 8*^#^	**<0**.**001**
FDd_avg_, %	29 ± 7	27 ± 7	28 ± 6	33 ± 8*^#^	**<0**.**001**
FDps, %	7 ± 5	5 ± 2	7 ± 5*	9 ± 7*	**<0**.**001**
ΔRA, °	−0.8 ± 42.7	−3.2 ± 25.1	10.2 ± 47.6	−9.4 ± 49.8	0.046
RSls_avg_, rev/s	−0.06 ± 0.77	−0.09 ± 0.69	0.08 ± 0.86	−0.15 ± 0.73	0.270
SFF, mL	73.0 ± 14.0	74.7 ± 12.9	71.6 ± 14.9	72.7 ± 14.2	0.500
SRF, mL	4.42 ± 4.24	1.68 ± 1.39	3.91 ± 3.29*	7.83 ± 4.77*^#^	**<0**.**001**
sFRR, %	5.9 ± 5.1	2.3 ± 1.7	5.2 ± 3.4*	10.5 ± 5.4*^#^	**<0**.**001**
Pulse wave velocity, m/s	4.3 ± 1.6	3.5 ± 0.8	3.8 ± 0.8	5.7 ± 2.0*^#^	**<0**.**001**
**CPET** ^§^					
PVO_2_, mL/kg/min	24 (19, 29)	26 (22, 32)	24 (19, 31)	23 (17, 25)*	**0**.**005**
METs	6.8 (5.5, 8.3)	7.3 (6.2, 9.1)	7.0 (5.4, 8.8)	6.4 (5.0, 7.2)*	**0**.**005**
% predicted PVO_2_, %	90 (74, 110)	89 (76, 110)	96 (72, 115)	92 (78, 101)	0.693
VE/VCO_2_ slope	26 (24, 29)	25 (24, 27)	26 (24, 29)*	28 (25, 30)*^#^	**<0**.**001**

Data were represented as mean ± SD or ^§^median (25th percentile, 75th percentile). AO, aorta; FD, flow displacement; FDd_avg_, average flow displacement during diastole; FDls_avg_, average flow displacement during late systole; FDps, flow displacement at peak systole; FDs_avg_, average flow displacement during systole; LV, left ventricle; LVEDV, left ventricular end-diastolic volume; LVESV, left ventricular end-systolic volume; LVSV, left ventricular stroke volume; METs, metabolic equivalents; ΔRA, the FD rotational angle change between end-systolic point and the point the flow angle stabilized after peak systole; RSls_avg_, average FD rotational speed after peak systole till end of systole; RV, right ventricle; RVEDV, right ventricular end-diastolic volume; RVESV, right ventricular end-systolic volume; RVSV, right ventricular stroke volume; SFF, systolic forward flow; sFRR, systolic flow reversal ratio; SRF, systolic retrograde flow; SV, stroke volume; PVO_2_, peak oxygen uptake; VE, minute ventilation; VCO_2_, carbon dioxide output. Late systole was defined after the peak systole to end systole. *P < 0.05 compared with Group one; ^#^*P* < 0.05 compared with Group two. No adjustment was made for multiple comparisons. Bold values denote statistical significance.

### Association between age and exercise capacity

PVO_2_, METs, and VE/VCO_2_ slope had significant differences across age subgroups, with Group three having the smallest values in PVO_2_ and METs and the greatest values in VE/VCO_2_ slope (*Table 1*). Moreover, PVO_2_ and METs were negatively associated with age (*r* = −0.214 and −0.213, *P* < 0.01), and VE/VCO_2_ was positively associated with age (*r* = 0.337, *P* < 0.0001). % predicted PVO_2_ was uncorrelated with age (*r* = 0.026, *P* = 0.739).

### Aortic flow dynamics in relation to age

**Figure 2:**
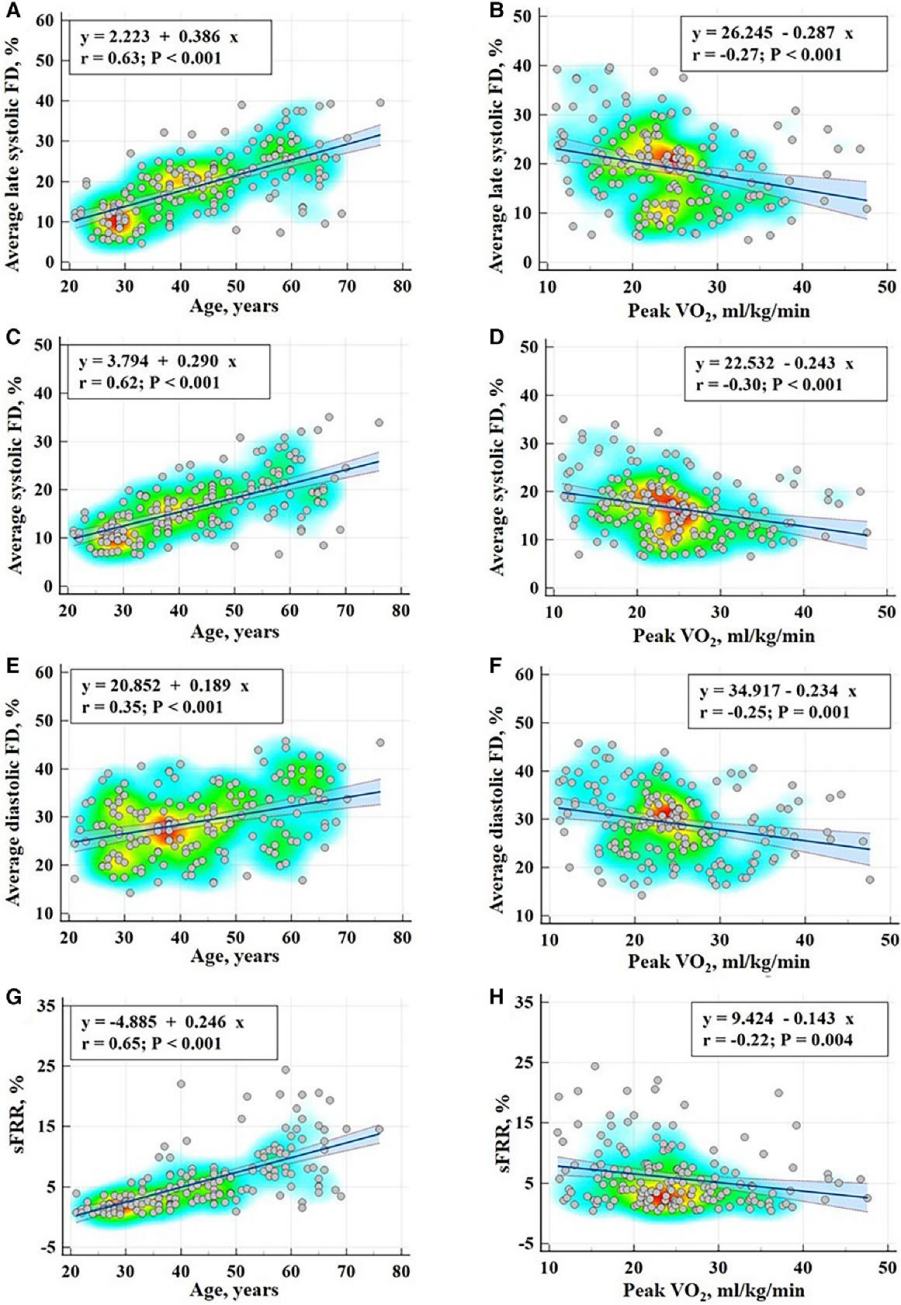
Scatterplots with regression lines, 95% confidence lines, and heat maps between flow parameters with age (left panel) and peak oxygen uptake (right panel) for average flow displacement during late systole (first row), systole (second row), and diastole (third row), and systolic flow reversal ratio (last row).

FDs_avg_, FDls_avg_, FDd_avg_, SRF, sFRR, and PWV had significant differences across the three age groups (*Table 1*). In particular, Group three had the largest values in these parameters compared with the other two age groups. Additionally, AO maximal and minimal areas significantly differed between the three age groups. The correlation coefficients of AO flow parameters and age are shown in *Table 2*. AO forward flow index and RAC were negatively associated with age (*r* = 0.173, *P* < 0.05 and *r* = −0.411, *P* < 0.0001), while AO backward flow index, AO maximal and minimal areas were positively associated with age (*r* = 0.303, 0.558, 0.627, *P* < 0.0001). In addition, FDs_avg_, FDls_avg_, FDd_avg_, FDps, SRF, sFRR, and PWV all increased with age (*r* = 0.623, 0.628, 0.353, 0.351, 0.590, 0.649, 0.598, all *P* < 0.0001). Scatterplots with regression lines, 95% confidence lines, and heat maps between FDs_avg_, FDls_avg_, FDd_avg_, sFRR, and age are given on the left panel of *Figure 2*.

**Table 2:** Correlation of 2D aortic flow parameters with age and cardiopulmonary exercise test parameters

	Age	PVO_2_	METs	VE/VCO_2_ slope
AO forward flow index, mL/m^2^	−0.173*	0.405***	0.403***	−0.056
AO backward flow index, mL/m^2^	0.303***	−0.057	−0.058	0.169*
AO maximal area, cm^2^	0.558***	0.016	0.014	0.267**
AO minimal area, cm^2^	0.627***	−0.035	−0.035	0.235**
Relative area change, %	−0.411***	0.081	0.078	−0.025
FDs_avg_, %	0.623***	−0.302***	−0.305***	0.313***
FDls_avg_, %	0.628***	−0.270**	−0.272**	0.303***
FDd_avg_, %	0.353***	−0.253**	−0.251**	0.056
FDps, %	0.351***	−0.111	−0.111	0.137
ΔRA, °	−0.081	0.087	0.085	0.019
RSls_avg_, rev/s	−0.046	0.068	0.067	0.046
SFF, mL	−0.002	0.317***	0.316***	0.027
SRF, mL	0.590***	−0.149*	−0.151*	0.326***
sFRR, %	0.649***	−0.219**	−0.221**	0.315***
Pulse wave velocity, m/s	0.598***	−0.161*	−0.159*	0.202**

* Significant level at *P* < 0.05; **Significant level at *P* < 0.01; ***Significant level at *P* < 0.0001.

### Aortic flow dynamics in relation to exercise capacity

**Figure 3:**
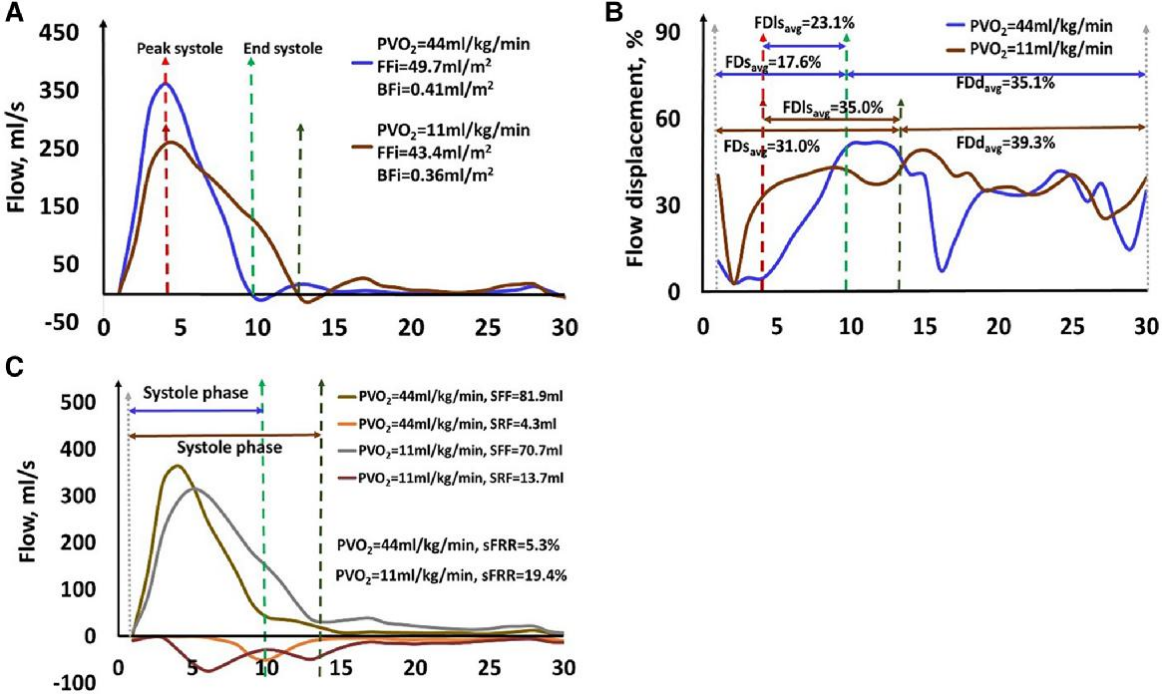
Example aortic flow curve (*A*), flow displacement curve (*B*), and flow reversal ratio (*C*) in a 65 year old male healthy subject with PVO_2_ = 44 mL/kg/min and a 67 year old female healthy subject with PVO_2_ = 11 mL/kg/min. The unit of the x-axis in each figure is the frame number.

Example curves for AO flow, FD, and FRR in two subjects (one with PVO_2_ > 20 mL/kg/min and one with PVO_2_ < 14 mL/kg/min) are given in *Figure 3*. The correlation coefficients of AO flow parameters with PVO_2_, METs, and VE/VCO_2_ slope are shown in *Table 2*. AO forward flow index was positively associated with PVO_2_, METs, and % predicted PVO_2_ (*r* = 0.405, 0.403, 0.304, *P* < 0.001). AO backward flow index, AO maximal, and minimal areas were positively associated with VE/VCO_2_ slope (*r* = 0.169, 0.267, 0.235, *P* < 0.05). FDs_avg_, FDls_avg_, FDd_avg_, SRF, sFRR, and PWV all decreased with PVO_2_ (*r* = −0.302, −0.270, −0.253, −0.149, −0.219, −0.161, all *P* < 0.05) and METs (*r* = −0.305, −0.272, −0.251, −0.151, −0.221, −0.159, all *P* < 0.05). FDs_avg_, FDls_avg_, SRF, sFRR, and PWV positively correlated with VE/VCO_2_ slope (*r* = 0.313, 0.303, 0.326, 0.315, 0.202, all *P* < 0.01), while only FDd_avg_ negatively correlated with % predicted PVO_2_ (*r* = −0.163, *P* < 0.05). Scatterplots with regression lines, 95% confidence lines, and heat maps between FDs_avg_, FDls_avg_, FDd_avg_, sFRR, and PVO_2_ are given on the right panel of *Figure 2*.

**Figure 4:**
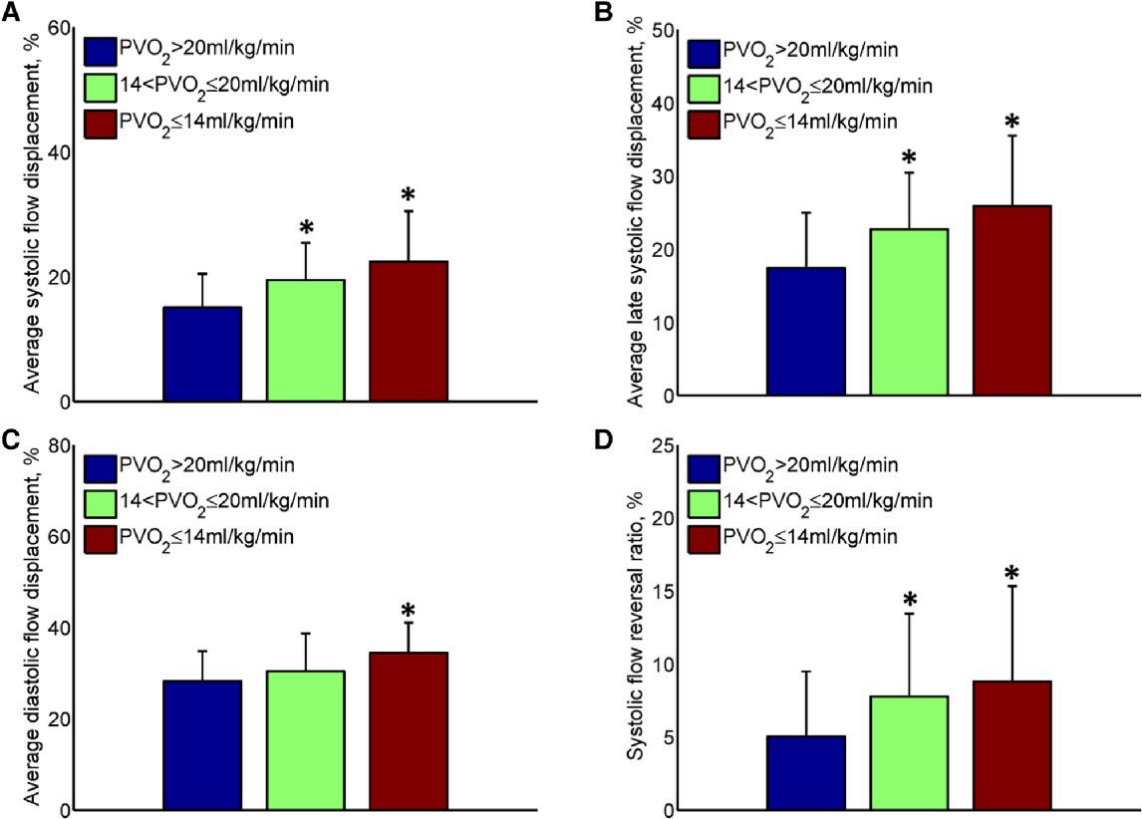
Differences in two-dimensional aortic flow parameters according to the subgroups based on peak oxygen uptake. (*A*) Average systolic flow displacement; (*B*) average late systolic flow displacement; (*C*) average diastolic flow displacement; (*D*) systolic flow reversal ratio. *P < 0.05 compared with PVO_2_ > 20 mL/kg/min. Error bars denote mean ± standard deviation.

A progressive increase in FDs_avg_, FDls_avg,_ FDd_avg_, and sFRR was observed with decreasing PVO_2_ (*Figure 4*). Both subjects with low- and intermediate-risk exercise capacity (14 < PVO_2_ ≤ 20 mL/kg/min) and subjects with high-risk exercise capacity (PVO_2_ ≤ 14 mL/kg/min) had significantly increased FDs_avg_, FDls_avg_, and sFRR compared with subjects with normal exercise capacity (PVO_2_ > 20 mL/kg/min) (*Figure 4*).

### Prediction of peak oxygen uptake by multi-variable regression model

A multi-variable linear regression model was constructed to predict PVO_2_ by LVEF, AO forward flow index, FDs_avg_, and the overall regression equation is as follows:

CMR-derived PVO_2_ = 20.729 + 0.443 * AO forward flow index—0.261 * FDs_avg_—0.172 * LVEF.

**Figure 5:**
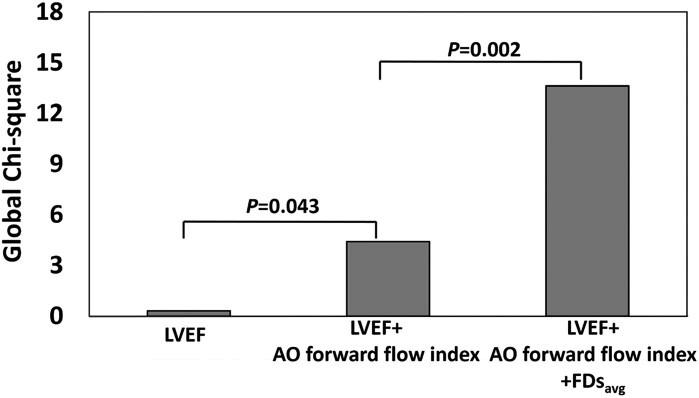
Incremental value of left ventricular ejection fraction, aortic forward flow index, and average flow displacement during systole for discriminating healthy subjects with PVO_2_ ≤ 14 mL/kg/min.

The CMR-derived PVO_2_ composite model was statistically significant (*R*^2^ = 0.245, *P* < 0.001) (*Table 3*). On ROC analysis, CMR-derived PVO_2_, FDs_avg_, FDd_avg_, FDls_avg_, SFF, and sFRR all had better discrimination vs. LVEF [area under the curve (AUC) 0.769, 0.742, 0.732, 0.727, 0.653, 0.583 vs. 0.560, respectively] for subjects with high-risk exercise capacity (PVO_2_ ≤ 14 mL/kg/min) (*Table 4*). On nested binary logistic regression analysis, adding FDs_avg_ to LVEF and AO forward flow index provided incremental value for detecting subjects with high-risk exercise capacity (*P* = 0.002) (*Figure 5*).

**Table 3:** Univariate and multi-variable linear regression analyses for peak oxygen uptake

	Univariate analysis		Stepwise	
	Coefficient (95% CI)	*P* value	Coefficient (95% CI)	*P* value
Age, yrs	−0.124 (−0.210, −0.037)	0.005	**—**	
**LVEF, %**	−0.189 (−0.352, −0.026)	0.023	**−0.172 (−0.319, −0.024)**	**0**.**023**
**AO forward flow index, mL/m^2^**	0.482 (0.316, 0.649)	<0.001	**0.443 (0.278, 0.609)**	**<0**.**001**
AO backward flow index, mL/m^2^	−0.778 (−2.878, 1.322)	0.466	**—**	
AO maximal area, cm^2^	0.073 (−0.627, 0.773)	0.837	**—**	
AO minimal area, cm^2^	−0.164 (−0.886, 0.558)	0.655	**—**	
Relative area change, %	0.038 (−0.034, 0.110)	0.298	**—**	
**FDs_avg_, %**	−0.376 (−0.557, −0.195)	<0.001	**−0.261 (−0.436, −0.086)**	**0**.**004**
FDls_avg_, %	−0.254 (−0.392, −0.115)	<0.001	**—**	
FDd_avg_, %	−0.273 (−0.433, −0.114)	0.001	**—**	
FDps, %	−0.159 (−0.378, 0.059)	0.153	**—**	
ΔRA, °	0.016 (−0.012, 0.043)	0.269	**—**	
RSls_avg_, rev/s	0.655 (−0.885, 2.195)	0.402	**—**	
SFF, mL	0.176 (0.096, 0.256)	<0.001	**—**	
SRF, mL	−0.272 (−0.548, 0.004)	0.053	**—**	
sFRR, %	−0.335 (−0.563, −0.107)	0.004	**—**	
Pulse wave velocity, m/s	−0.762 (−1.482, −0.042)	0.038	**—**	
Coefficient	—	—	20.729 (8.866, 32.593)	**0**.**001**
R-squared, multi-variable				0.245

Bold values denote statistical significance.

**Table 4:** Utility of aortic flow parameters to detect PVO_2_ ≤ 14 mL/kg/min in healthy subjects with area under the curve, p value, sensitivity, specificity, and threshold

	AUC (95% CI^a^)	*P*	Sensitivity (95% CI)	Specificity (95% CI)	Threshold
CMR-derived PVO_2_	0.769 (0.670, 0.861)	**<0**.**001**	100 (75, 100)	58 (50, 66)	24.0
FDs_avg_, %	0.742 (0.574, 0.909)	**0**.**005**	77 (46, 95)	69 (61, 75)	18.62
FDd_avg_, %	0.732 (0.594, 0.869)	**0**.**001**	85 (55, 98)	58 (50, 66)	29.61
FDls_avg_, %	0.727 (0.565, 0.888)	**0**.**006**	69 (39, 91)	76 (69, 83)	23.49
sFRR, %	0.653 (0.490, 0.816)	0.066	39 (14, 69)	90 (84, 94)	11.73
SRF, mL	0.627 (0.464, 0.790)	0.128	39 (14, 68)	86 (79, 91)	7.55
SFF, mL	0.583 (0.438, 0.727)	0.262	100 (75, 100)	22 (16, 29)	83.8
LVEF, %	0.560 (0.417, 0.704)	0.411	92 (64 100)	30 (23, 37)	58

CI, confidence interval; CMR-derived PVO_2_, variable from multi-variable linear regression model (CMR-derived PVO_2_ = 20.729 + 0.443 * aortic forward flow index—0.261 * FDs_avg_—0.172 * LVEF). Bold values denote statistical significance.

a AUC ± 1.96 standard error.

### Reproducibility

**Figure 6:**
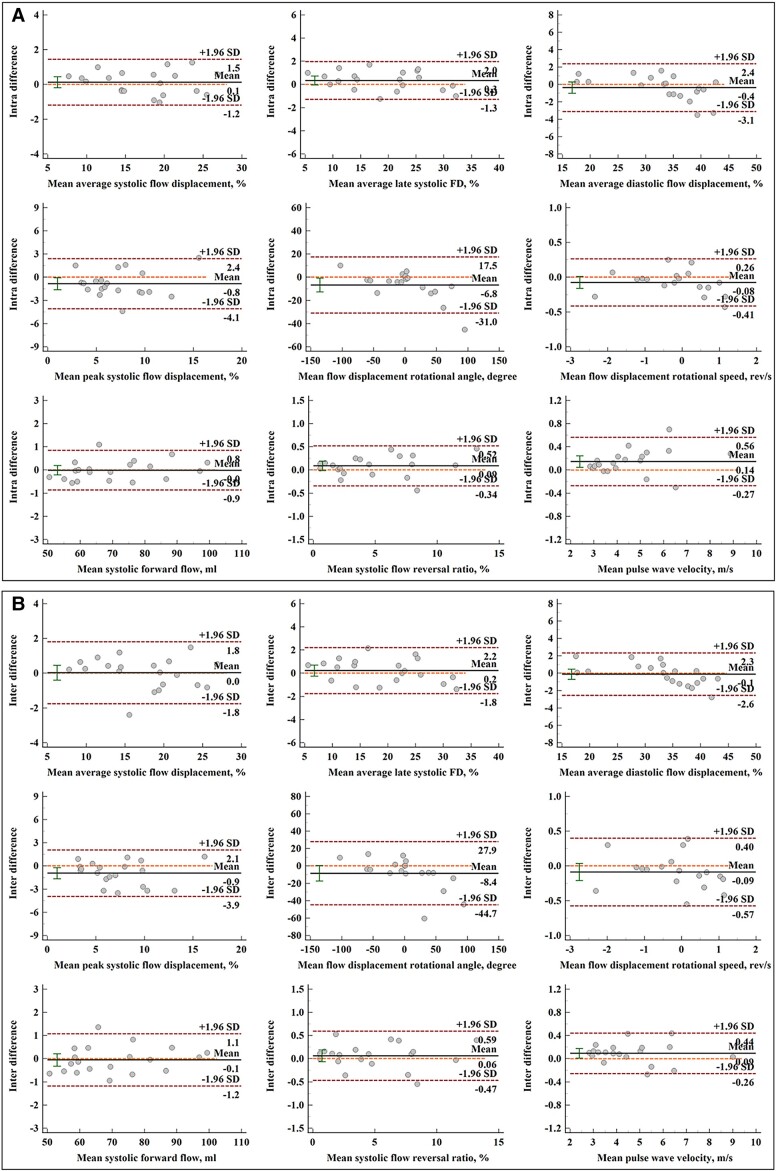
Bland–Altman plots for aortic blood flow components. (*A*) Bland–Altman analysis of intraobserver repeated measurements; (*B*) Bland–Altman analysis of interobserver repeated measurements. From left to right, top to bottom: average flow displacement during systole, late systole and diastole, peak systolic flow displacement, rotational angle, rotational speed, systolic forward flow, systolic flow reversal ratio, and pulse wave velocity.

The reproducibility results of AO blood flow parameters in 20 subjects are tabulated in *Table 5*. Both intra- and interobserver had excellent ICC coefficients (all > 0.935, *P* < 0.001). Mean differences of intra- and interobserver measurements were small with good limits of agreement, and Bland–Altman plots of intra- and interobserver measurements were provided in *Figure 6*. Coefficients of variation for intra- and interobserver reproducibility were all ≤4% if applicable (*Table 5*).

**Table 5:** Intra- and interobserver agreement

	Mean difference	ICC (95% CI)	*P*	CV
Intraobserver				
AO forward flow, mL	−0.06 ± 0.64	1.000 (0.999, 1.000)	<0.001	0.61
AO backward flow, mL	0.08 ± 0.07	0.997 (0.993, 0.999)	<0.001	/
FDs_avg_, %	−0.85 ± 1.66	0.996 (0.991, 0.999)	<0.001	2.72
FDls_avg_, %	0.13 ± 0.67	0.997 (0.993, 0.999)	<0.001	3.25
FDd_avg_, %	0.34 ± 0.83	0.991 (0.978, 0.997)	<0.001	3.01
FDps, %	−0.37 ± 1.40	0.935 (0.836, 0.974)	<0.001	/
ΔRA, °	−6.8 ± 12.4	0.984 (0.959, 0.994)	<0.001	/
RSls_avg_, rev/s	−0.08 ± 0.17	0.993 (0.980, 0.997)	<0.001	/
SFF, mL	−0.01 ± 0.44	1.000 (0.999, 1.000)	<0.001	0.43
SRF, mL	0.07 ± 0.17	0.999 (0.998, 1.000)	<0.001	3.57
sFRR, %	0.09 ± 0.22	0.999 (0.998, 1.000)	<0.001	3.33
Pulse wave velocity, m/s	0.15 ± 0.21	0.995 (0.988, 0.998)	<0.001	3.92
Interobserver				
AO forward flow, mL	−0.15 ± 0.71	0.999 (0.998, 1.000)	<0.001	0.70
AO backward flow, mL	0.07 ± 0.10	0.996 (0.989, 0.998)	<0.001	/
FDs_avg_, %	0.03 ± 0.91	0.993 (0.983, 0.997)	<0.001	3.60
FDls_avg_, %	0.22 ± 1.02	0.996 (0.990, 0.998)	<0.001	3.76
FDd_avg_, %	−0.12 ± 1.25	0.993 (0.983, 0.997)	<0.001	2.61
FDps, %	−0.94 ± 1.53	0.950 (0.874, 0.980)	<0.001	/
ΔRA, °	−8.4 ± 18.5	0.966 (0.911, 0.987)	<0.001	/
RSls_avg_, rev/s	−0.09 ± 0.25	0.985 (0.960, 0.994)	<0.001	/
SFF, mL	−0.05 ± 0.58	1.000 (0.999, 1.000)	<0.001	0.57
SRF, mL	0.05 ± 0.19	0.999 (0.997, 0.999)	<0.001	4.00
sFRR, %	0.06 ± 0.27	0.999 (0.997, 0.999)	<0.001	3.88
Pulse wave velocity, m/s	0.09 ± 0.18	0.997 (0.992, 0.999)	<0.001	3.03

ICC, intraclass correlation coefficient; CV, coefficients of variation. ‘/’ CV was not calculated if the mean value was near 0.

## Discussion

This is the first study exploring the significance of AO flow changes and their association with functional exercise-related outcomes in the context of aging without specific cardiovascular disease. As expected, functional exercise-related outcomes progressively declined with aging. AO FD during systole, which suggests a more eccentric ascending aorta flow profile, increased with age. In addition, we observed that the systolic FRR increased with age, implying a reduction in AO conduit function. Most importantly, in multi-variable analysis, exercise performance measured by PVO_2_ demonstrated an independent association with AO forward flow index and average systolic FD.

Whilst numerous previous studies have demonstrated abnormal AO flow parameters in pathological states such as bicuspid AO valve and AO aneurysm,^16,18,26,27,28^ this is the first study to show that abnormal flow parameters correlate with decreased quantified exercise functional capacity without specific pathology, in particular, AO valve stenosis. Notably, both FD and systolic FRR had a better association with PVO_2_ than established AO stiffness parameters like PWV. The plausible explanation for this finding is that FD is a marker of flow eccentricity which results in turbulent flow in the ascending aorta leading to less efficient blood flow transportation. This makes the overall cardiovascular system less effective and reduces the exercise capacity directly. In addition, sFRR results in a direct loss of forward flow by reducing the conduit function of the ascending AO root during systole. This will result in reduced perfusion and reduced oxygen delivery to tissues, affecting both the PVO_2_ and METs.

Several studies investigating AO root dilatation have already studied systolic FD.^17,29,30^ Not only is FD associated with AO dilatation, but it can also predict further dilatation at 1-year follow-up,^18^ and is significantly linked to LV remodelling in patients with AO stenosis.^31^ Similar observations were made by Albarran *et al.* in pre-clinical pig models.^29^ These studies demonstrate the importance of this AO flow parameter which indicates not only eccentricity of flow and its associated imbalances of wall shear stress leading to further AO dilatation but also turbulent flow resulting in energy loss and reduced functional capacity.

**Figure 7:**
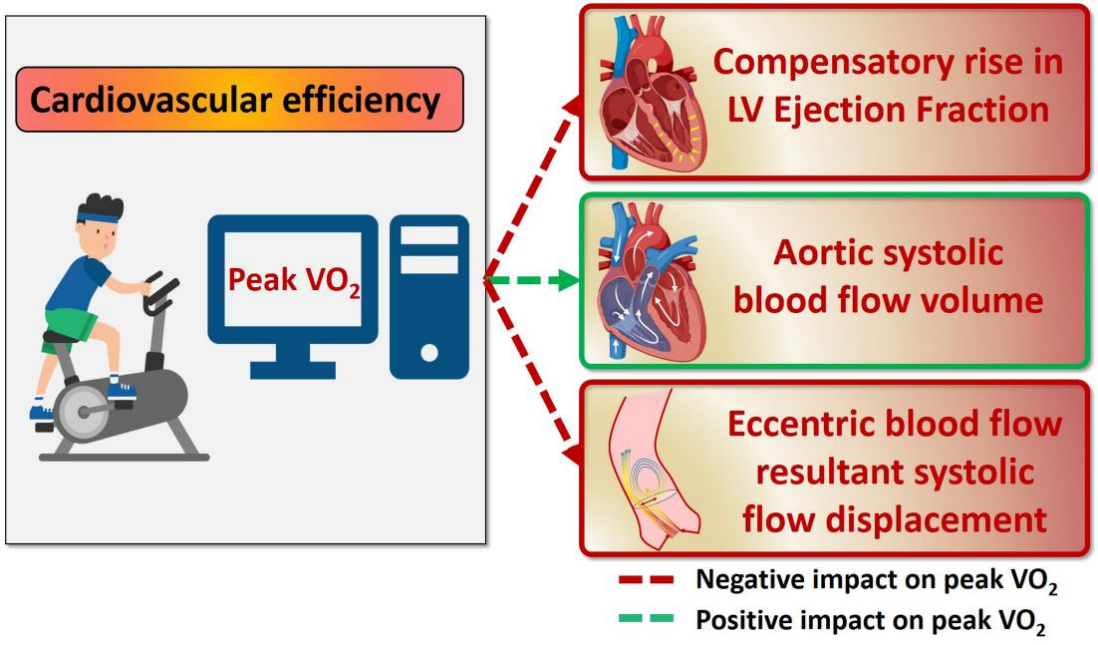
Central illustration demonstrating cardiovascular magnetic resonance-derived physiological model for estimating peak oxygen uptake non-invasively without exercise.

In our cohort of community healthy volunteers with largely normal heart function, we were able to predict exercise capacity using only imaging parameters. In our multi-variable regression model, PVO_2_ was positively associated with AO forward flow index and negatively associated with systolic FD and LVEF, which characterize resultant stroke volume (SV), turbulent blood flow, and ventricular contraction, respectively (*Figure 7*). Two important implications are apparent. First, the prediction is age-insensitive, which allows for functional the capacity to be estimated using only imaging. Second, the negative association between exercise capacity and LVEF needs further explanation. While reduced LVEF can certainly impair exercise capacity, recent research in community-based populations, such as our study, suggests that those participants with supranormal LVEF >65%^32^ or >70%^33^ may in fact experience worse cardiovascular outcomes, especially those with low stroke volumes.^32^ Therefore, our model predictions are consistent with and offer unique mechanistic insights into, this emerging evidence.

### Clinical applicability

CPET provides a comprehensive pathophysiological evaluation of patients’ exercise limitation and dyspnoea, but is time-intensive and not universally available due to lack of expertise in the carrying out of CPET.^34^ CMR offers multi-parametric assessment, and exercise capacity prediction is a valuable complement to structural and functional cardiovascular imaging surveillance. The multi-variable model to estimate PVO_2_, which incorporates not only LVEF but also FDs_avg_ and AO forward flow indexed to BSA, would be amenable to high-throughput analysis and could be easily added to current CMR reporting platforms. Moreover, FD and sFRR do not require advanced computational four-dimensional flow analysis and can be easily applied to 2D PC, making them more widely applicable. It is already established that ventriculo-arterial coupling (VAC) is directly linked with PVO_2_.^35^ Even though in this study, we are not directly measuring the actual VAC, we plausibly propose that the CMR-derived model of PVO_2_ is representative of VAC. This is because it incorporates ventricular function and arterial flow dynamics associated with ascending AO efficiency. Since VAC ultimately defines the performance and efficiency of the cardiovascular system, the analysis of the interaction between the heart and the arterial system could offer a broader perspective of the haemodynamic disorders associated with common conditions, such as heart failure. Moreover, this analysis could also provide valuable information about their pathophysiological mechanisms and may help to determine the best therapeutic strategy to correct them.

### Limitations

Limitations of the study include its observational nature, as at present, no intervention exists to improve AO flow parameters except perhaps exercise training. As such, it is limited to demonstrating correlations rather than causality. The very elderly population is not well represented in this study. This group would likely be challenging to study due to other mobility issues limiting the ability to perform CPET. Potential selection bias exists, with those who are able to perform CPET being likely to have more normal AO flows, although this would be expected to blunt rather than enhance the present results. Our regression model for prediction of exercise capacity was based on a community cohort of healthy volunteers with largely normal heart function, and may not be generalizable to patients, e.g. those with heart failure with reduced ejection fraction or other pathological conditions. Nevertheless, its mechanical basis, which encompasses resultant SV, turbulent blood flow, and ejection fraction, is physiologically plausible and, importantly, feasibly assessable using only simple imaging methods. The study does not have long-term follow-up data and, therefore, cannot comment on whether AO haemodynamics contribute to morbidity and mortality. However, it would be expected that better exercise performance would correlate with long-term outcomes. It is widely known that when the acquisition plane of 2D PC is not positioned exactly orthogonal to the direction of flow in the aorta, and the peak velocity is underestimated,^36^ while its effect on the flow calculation should be minor as mentioned in the previous study.^37^ In our study, 2D PC ascending aorta images were acquired at an orthogonal plane just above the sinotubular junction. This plane also approximates a plane that transects the descending thoracic AO orthogonally in normal anatomy. It should be noted that AO flow parameters might be different, especially in the descending thoracic aorta, if the acquisition plane is placed at other locations or with altered anatomy. AO FD, FRR, and PWV can also be calculated in a similar way using 4D flow CMR, and some more advanced descriptors, such as turbulent kinetic energy,^38^ viscous energy loss,^39^ can also be obtained in the aorta with 4D flow CMR. Unlike conventional 2D PC imaging, 4D flow CMR requires additional scanning time and dedicated software/algorithm for post-processing, and shall follow the latest updated consensus.^40^

## Conclusions

AO flow changes are associated with age and reduced functional performance as assessed by 2D PC CMR. AO forward flow indexed to BSA, LV ejection fraction, and average systolic FD can be used to predict exercise capacity measured PVO_2_.

## Supplementary Material

oead079_Supplementary_Data

## Data Availability

The datasets used and/or analysed during the current study are available from the corresponding author upon reasonable request.

## Lead author biography

Dr. Xiaodan Zhao graduated from Nanyang Technological University Singapore with major in a Computational Mathematics, and currently works at National Heart Research Institute Singapore, National Heart Centre Singapore. Her research focus is to develop CMR image markers and translate them into patient care.
