# Corrigendum to: Aortic flow is associated with aging and exercise capacity

**DOI:** 10.1093/ehjopen/oead105

**Published:** 2023-10-26

This is a corrigendum to: Xiaodan Zhao, Pankaj Garg, Hosamadin Assadi, Ru-San Tan, Ping Chai, Tee Joo Yeo, Gareth Matthews, Zia Mehmood, Shuang Leng, Jennifer Ann Bryant, Lynette L S Teo, Ching Ching Ong, James W Yip, Ju Le Tan, Rob J van der Geest, Liang Zhong, Aortic flow is associated with aging and exercise capacity, European Heart Journal Open, Volume 3, Issue 4, July 2023, oead079, https://doi.org/10.1093/ehjopen/oead079

The graphical abstract of this manuscript has been corrected.

The revised graphical abstract includes a new image of the CMR scanner, a new CPET image, and several abbreviations which were omitted in the original publication (CMR, FD, and AO forward flow index).

