# Supplementary material for: Aortic flow is associated with aging and exercise capacity
Source: Eur Heart J Open. 2023 Aug 26;3(4):oead079. doi: 10.1093/ehjopen/oead079 (PMC10460199; doi:10.1093/ehjopen/oead079)
Supplement: oead079_Supplementary_Data [file oead079_supplementary_data.docx]

**Supplementary Table 1.** Acquisition parameters of cine and 2D phase-contrast CMR imaging in two centres.

| Vendor | Philips | Siemens |
| --- | --- | --- |
| Magnetic field strength | 3.0T | 1.5T |
| Pulse sequence | Spoiled gradient echo | Spoiled gradient echo |
| **Cine images** | | |
| TR/TE (ms) | 2.8/1.4 | 3.4/1.3 |
| Flip angle (°) | 45 | 72 |
| Field of view (mm^2^) | 320 x 320 | 320 x 260 |
| Slice thickness, mm | 8 | 8 |
| Cardiac phases | 30 | 30 |
| **2D phase-contrast images** | | |
| Field of view (FOV) (mm^2^) | 320 × 320 | 233 × 340 |
| Percent phase FOV, % | 94.44 | 68.75 |
| Bandwidth, Hz/pixel | 723 | 455 |
| TR/TE (ms) | 4.23/2.63 | 20.36/2.8 |
| Flip angle (°) | 10 | 20 |
| Cardiac phases | 30 | 30 |
| VENC (cm/s) | 150 | 150 |

*2D* two-dimensional, *CMR* cardiovascular magnetic resonance, *ECG* electrocardiogram, *SENSE* sensitivity encoding, *TR* repetition time, *TE* echo time, *VENC* velocity encoding.

**Supplementary Table 2.** Comparison of 2D aortic flow and cardiopulmonary exercise test (CPET) parameters between male and female subjects.

|  | **Male (n = 96)** | **Female (n = 73)** | ***P*** |
| --- | --- | --- | --- |
| **2D aortic flow parameters** |  |  |  |
| AO forward flow index, ml/m^2^ | 43.4 ± 7.1 | 41.7 ± 5.5 | 0.086 |
| AO backward flow index, ml/m^2^ | 0.68 ± 0.54 | 0.38 ± 0.55 | **0.001** |
| AO max area, mm^2^ | 7.6 ± 1.7 | 6.5 ± 1.5 | **<0.001** |
| AO min area, mm^2^ | 6.2 ± 1.6 | 4.9 ± 1.5 | **<0.001** |
| Relative area change, % | 24 ± 11 | 35 ± 20 | **<0.001** |
| FDs_avg_, % | 15 ± 6 | 18 ± 7 | **0.003** |
| FDls_avg_, % | 18 ± 8 | 21 ± 9 | **0.007** |
| FDd_avg_, % | 29 ± 7 | 30 ± 7 | 0.422 |
| FDps, % | 6 ± 4 | 8 ± 7 | **0.046** |
| ΔRA, ° | -1.5 ± 40.2 | 0.2 ± 46.0 | 0.807 |
| RSls_avg_, rev/s | -0.06 ± 0.83 | -0.05 ± 0.69 | 0.897 |
| SFF, ml | 78.7 ± 13.2 | 65.5 ± 11.2 | **<0.001** |
| SRF, ml | 4.44 ± 4.07 | 4.39 ± 4.49 | 0.943 |
| sFRR, % | 5.6 ± 4.7 | 6.3 ± 5.5 | 0.362 |
| Pulse wave velocity, m/s | 4.3 ± 1.4 | 4.1 ± 1.9 | 0.191 |
| **CPET**^§^ |  |  |  |
| PVO_2_, ml/kg/min | 26 (22, 34) | 21 (17, 25) | **<0.001** |
| METs | 7.4 (6.2, 9.6) | 6.0 (4.8, 7.2) | **<0.001** |
| % predicted PVO_2_, % | 92 (80, 113) | 87 (72, 104) | 0.122 |
| VE/VCO_2_ slope | 27 (24, 29) | 26 (25, 28) | 0.931 |

Data were represented as mean ± SD or ^§^median (25^th^ percentile, 75^th^ percentile). *AO* aorta, *EDV* end-diastolic volume, *ESV* end-systolic volume, *FD* flow displacement, *FDd_avg_* average flow displacement during diastole, *FDls_avg_* average flow displacement during late systole, *FDps* flow displacement at peak systole, *FDs_avg_* average flow displacement during systole, *LV* left ventricle, *METs* metabolic equivalents, *ΔRA* the FD rotational angle change between end-systolic point and the point the flow angle stabilised after peak systole, *RSls_avg_* average FD rotational speed after peak systole till end of systole, *RV* right ventricle, *SFF* systolic forward flow, s*FRR* systolic flow reversal ratio, *SRF* systolic retrograde flow, *SV*  stroke volume, *PVO_2_* peak oxygen uptake, *VE* minute ventilation, *VCO_2_* carbon dioxide output. Late systole was defined after the peak systole to end systole.


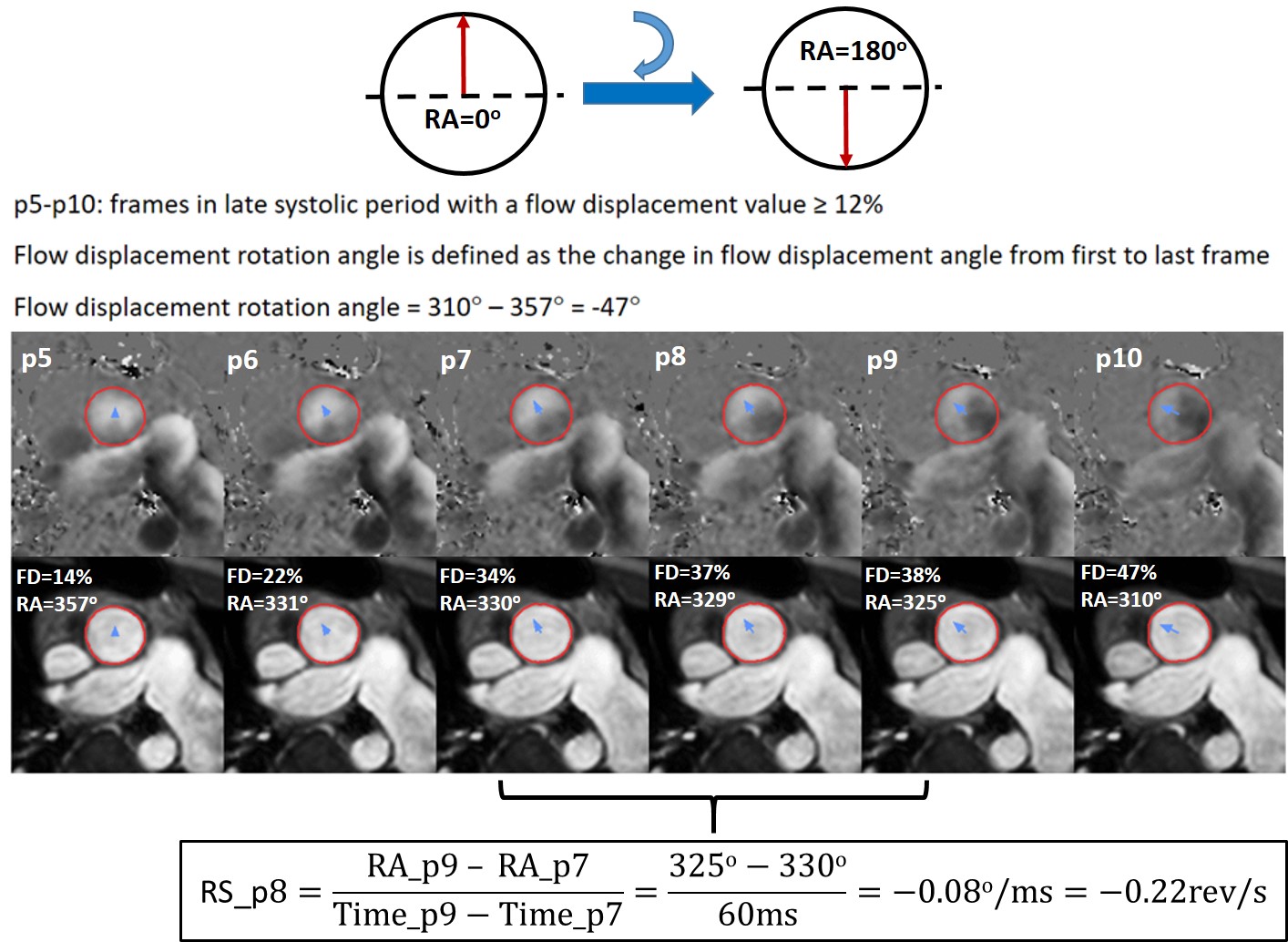


**Figure S1**. Illustration of how to calculate the ΔRA and rotational speed (RS) at a given phase. Flow displacement rotational angle (RA) is the angle formed by the anterior-pointing radius (12 o'clock position) and the line segment connecting the vessel centre point and the centre-of-velocity of the forward flow in the ascending aortic cross-section on 2D PC image at every phase of the heart cycle. For a given phase, RS was derived as the time derivative of the rotational angle using the next and previous phases, and was only computed for those phases with FD >12% for the current phase as well as for the previous and next phases.

**Extended Methods**

**2D phase-contrast flow analysis**

The detailed calculation of 2D aortic flow parameters – flow displacement, flow displacement rotational angle, and pulse wave velocity were given below.

- Flow displacement (FD) was calculated as the distance between the vessel centre point and the centre-of-velocity of the forward flow and was normalised to overall vessel size for each cardiac phase.^1,2^ The centre point was computed as the centre of gravity of the contour points; the centre-of-velocity was calculated as the average position of pixels weighted by the velocity information within the defined aortic contour, and the vessel size was computed as the average vessel radius assuming the defined aortic contour being circular.^1^
- Flow displacement rotational angle (RA) is the angle subtended by a line segment connecting the centre-of-velocity of the forward flow and and the anterior-pointing radius (12 o'clock position) in the ascending aortic cross-section on each 2D PC image at every phase of the heart cycle. To ensure consistency of the measurement across subjects, the 2D PC images were acquired using standardized slice positioning. RA was defined as zero when the vector was pointing anteriorly (12 o’clock position), and clockwise turned to 180 degrees when pointing posteriorly (6 o’clock position). A figure illustrating on the definition of RA at 0 and 180 degree and calculation of ΔRA is given in **Supplementary material online, Figure S1**. The aortic blood flow is mainly laminar in early systole,^3^ and the position of the centre-of-velocity of the forward flow within the defined aortic contour approximates the vessel centre point, resulting in negligible flow displacement. At low flow displacements, both the centre-of-velocity of the forward flow and the vessel centre point become sensitive to errors in the aortic contour definition. A slight modification of aortic contour can displace the location of the centre-of-velocity of the forward flow causing significant change in the rotational angle. Hence, it was necessary to use a threshold value of FD, below which the rotational angle is assumed to be zero, to minimize the effect of noise. We chose a FD=12% threshold based on results of our bench testing to circumvent this issue in the current study.
- Pulse wave velocity (PWV) was calculated as the ratio of distance and transit time between ascending to descending aorta. In MASS, a “candy-cane” image encompassing the ascending aorta, aortic arch and descending aorta was 3D reconstructed from a stack of parallel transverse thoracic scout images using a three-point localization technique based on the following landmarks: centres of ascending and descending aorta, and the arch. Next, the imaging plane of the 2D PC aortic flow acquisition, which transected the ascending and descending aorta, was co-registered on the reconstructed 3D aortic image, thereby establishing on the latter, the starting and ending points corresponding to the precise locations where the ascending and descending aorta through-plane velocities were measured. The arch length was then traced as the intraaortic centreline path connecting these two locations. The transit time was calculated as the time difference between the points where the ascending and descending aorta flow waveforms reached half maxima of their peak velocities (**Fig 1. (G)**).^4^

References

1. Sigovan M, Hope MD, Dyverfeldt P, Saloner D. Comparison of four-dimensional flow parameters for quantification of flow eccentricity in the ascending aorta. *J Magn Reson Imaging* 2011;34:1226-30.
2. Burris NS, Sigovan M, Knauer HA, Tseng EE, Saloner D, Hope MD. Systolic flow displacement correlates with future ascending aortic growth in patients with bicuspid aortic valves undergoing magnetic resonance surveillance. *Invest Radiol* 2014;49:635–9.
3. Bissell MM, Dall'Armellina E, Choudhury RP. Flow vortices in the aortic root: in vivo 4D-MRI confirms predictions of Leonardo da Vinci. *Eur Heart J* 2014;35:1344.
4. Groenink M, de Roos A, Mulder BJ, Verbeeten B Jr, Timmermans J, Zwinderman AH, et al. Biophysical properties of the normal-sized aorta in patients with Marfan syndrome: evaluation with MR flow mapping. *Radiology* 2001;219:535-40.
